# Frequency Splitting Analysis and Compensation Method for Inductive Wireless Powering of Implantable Biosensors

**DOI:** 10.3390/s16081229

**Published:** 2016-08-04

**Authors:** Matthew Schormans, Virgilio Valente, Andreas Demosthenous

**Affiliations:** Department of Electronic and Electrical Engineering, University College London, London WC1E 7JE, UK; v.valente@ucl.ac.uk (V.V.); a.demosthenous@ucl.ac.uk (A.D.)

**Keywords:** frequency splitting, frequency tuning, implantable biosensors, inductive link, inductive powering, medical implants, wireless power transfer

## Abstract

Inductive powering for implanted medical devices, such as implantable biosensors, is a safe and effective technique that allows power to be delivered to implants wirelessly, avoiding the use of transcutaneous wires or implanted batteries. Wireless powering is very sensitive to a number of link parameters, including coil distance, alignment, shape, and load conditions. The optimum drive frequency of an inductive link varies depending on the coil spacing and load. This paper presents an optimum frequency tracking (OFT) method, in which an inductive power link is driven at a frequency that is maintained at an optimum value to ensure that the link is working at resonance, and the output voltage is maximised. The method is shown to provide significant improvements in maintained secondary voltage and system efficiency for a range of loads when the link is overcoupled. The OFT method does not require the use of variable capacitors or inductors. When tested at frequencies around a nominal frequency of 5 MHz, the OFT method provides up to a twofold efficiency improvement compared to a fixed frequency drive. The system can be readily interfaced with passive implants or implantable biosensors, and lends itself to interfacing with designs such as distributed implanted sensor networks, where each implant is operating at a different frequency.

## 1. Introduction

Short-range wireless power transfer (WPT) by means of inductive coupling is a safe and established technique, extensively used to transfer power to implanted medical devices (IMDs). WPT avoids the use of transcutaneous wires in applications where implanted batteries do not represent a viable alternative, for example, in high-density visual and brain implants [1,2,3,4], neuromuscular interfaces [5,6], and implanted biosensors [7,8,9].

Inductive links are based on weak coupling between an external transmitter coil and an implanted receiver coil optimized for operation at a specific resonant frequency and distance between the coils. Deviations in coil separation, alignment, shape, and load conditions greatly affect the link gain, resulting in significant variations in the power delivered to the implant. In particular, a phenomenon known as frequency splitting occurs in WPT systems when the coils are driven in the overcoupled regime [10]. Under these conditions, the link gain shows two peaks at different frequencies, which deviate from the tuned resonant frequency. Fixed-frequency WPT systems therefore suffer from dramatic changes in the output voltage as the relative distance between the coils varies. In order to maintain high system efficiency and constant supply voltage in the implant despite varying conditions, adaptive methods of tracking the splitting frequency and providing coupling-insensitive gain are required [11].

In fixed frequency WPT systems, uniform power delivery is commonly achieved by sensing the DC load voltage, transmitting its value to the transmitter via back-telemetry, and appropriately adjusting the supply voltage at the transmitter [12,13,14]. This method is suitable as long as the transmitter and receiver units are working close to resonance. Under overcoupled conditions, a significant increase in primary supply voltage is required to compensate for the occurrence of frequency splitting. This leads to a consequent drop in system efficiency. Alternative methods rely on careful optimization of the coil design to allow for coupling insensitive power transfer [15], but fail to address deviations from optimal operation arising from drifts in coil electrical parameters and geometric deformations. A number of adaptive systems have been described that accomplish power regulation in the secondary by electronically varying inductance [16,17] or capacitance [18] in either the primary or the secondary tank circuit. However, component variations and drift may further reduce the link efficiency, in particular where flexible and stretchable coils are used, whose parameters may fall outside the available compensation range. In addition, the achievable frequency resolution is determined by the number of taps in the tuning circuit, which limits the improvement in system efficiency that can be obtained. A triple-loop automatic tuning system including transmitter and receiver tuning and power control is reported in [19]. In addition to the limited resolution available, this system is only suitable for operation at a fixed frequency, and relies heavily on back telemetry to inform local control units.

An alternative approach to these methods is to directly control the drive frequency of the WPT system [20]. This has the benefit of requiring no variable link components, only a variable frequency synthesizer. There are several benefits to this approach. Firstly, the design of the power transmitter can be considered largely separately from the design of the coils, as long as the frequency synthesizer has a wide range. Secondly, a variable frequency synthesizer can be integrated on chip, saving valuable space in a compact transmitter compared to variable link components. Additionally, this approach requires no back-telemetry, as the link state is measured and maintained from the primary side. For these reasons, control of the drive frequency is the basis for the method described in this paper.

This paper presents an active compensation method for frequency splitting, based on optimum frequency tracking (OFT) in the overcoupled regime [21,22]. It can operate over a wide frequency range, and does not require the use of variable capacitors or inductors. A simple analytical derivation of the link gain is presented, that accounts for different coupling and load conditions. Theoretical link gain profiles are derived for a series-parallel link operating at fixed and variable frequencies. A compact closed-loop automatic frequency tuning system is presented. The link efficiency is optimized by sensing the phase between the primary voltage and current, and adjusting the frequency of the transmitter driver. This implementation uses a custom integrated, high-power Class-D amplifier with an on-chip frequency synthesizer [23], which allows for very fine frequency resolution to be obtained.

The rest of the paper is organised as follows. Section 2 presents some fundamentals of inductive link design and a simple theoretical analysis of link gain. Section 3 describes the proposed system. Section 4 describes the testing methodology and the measured results from employing the OFT compensation method. Section 5 concludes the paper, and provides a comparison with other work.

## 2. Inductive Link Theory

### 2.1. Inductive Link Fundamentals

**Figure 1:**
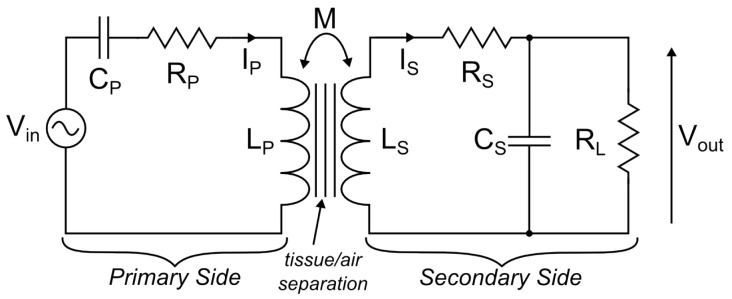
Idealised series-parallel inductive link model.

A series-parallel model of a resonant inductive link is shown in Figure 1. This configuration allows the input to be driven with an AC voltage source $V_{\text{in}}$, and the output can be considered a voltage source $V_{\text{out}}$ as a result of current in the output load $R_{L}$.

The primary and secondary sides consist of the coil inductances, $L_{P}$ and $L_{S}$, the coil series resistances, $R_{P}$ and $R_{S}$, and the tuning capacitors, $C_{P}$ and $C_{S}$. *M* is the mutual inductance between the coils, which defines the coupling coefficient, $k=M/\sqrt{L_{P}L_{S}}$. $R_{L}$ represents the equivalent load presented by the implant at the secondary. The tuned resonant frequency of both sides is defined in Equation (1):

(1) $$\omega_{0}=\frac{1}{\sqrt{L_{P}C_{P}}}=\frac{1}{\sqrt{L_{S}C_{S}}}$$

The link gain, $A=V_{\text{out}}/V_{\text{in}}$, can be determined as follows, in accordance with [24]. Firstly, the secondary circuit can be considered as a reflected impedance $Z_{\text{refl}}$, presented in series with the primary circuit:

(2) $$Z_{\text{refl}}=\frac{{\left(\omega M\right)}^{2}}{Z_{S}}$$

where $Z_{S}$ is the equivalent impedance of the secondary side of the link, and *ω* is the drive frequency. Considering the effect of $Z_{\text{refl}}$, the current in the primary side is therefore:

(3) $$I_{P}=\frac{V_{\text{in}}}{Z_{P}+\frac{{\left(\omega M\right)}^{2}}{Z_{S}}}$$

where $Z_{P}$ is the equivalent impedance of the primary circuit (not including $Z_{\text{refl}}$). The voltage induced in the secondary by the primary circuit is $-j\omega MI_{P}$, and from Equation (3):

(4) $$I_{S}=\frac{-j\omega MV_{\text{in}}}{Z_{P}Z_{S}+{\left(\omega M\right)}^{2}}$$

Equation (4) defines the output voltage $V_{\text{out}}$:

(5) $$V_{\text{out}}=\frac{I_{S}}{j\omega C_{S}+\frac{1}{R_{L}}}$$

Therefore, since in the series-parallel configuration $V_{\text{out}}$ appears across $C_{S}\left|\right|R_{L}$, the gain *A* can be written as follows:

(6) $$A=\frac{V_{\text{out}}}{V_{\text{in}}}=\frac{-j\omega M}{\left(Z_{P}Z_{S}+{\left(\omega M\right)}^{2}\right)\left(j\omega C_{S}+\frac{1}{R_{L}}\right)}=\frac{-j\omega k\sqrt{L_{P}L_{S}}}{\left(Z_{P}Z_{S}+\omega^{2}k^{2}L_{P}L_{S}\right)\left(j\omega C_{S}+\frac{1}{R_{L}}\right)}$$

Equation (6) can be greatly simplified by assuming an ideal resonant condition of $\omega =\omega_{0}$. However, this assumption does not hold true when the link coils are overcoupled, where the link will resonate at an optimum frequency $\omega_{\text{opt}}\neq \omega_{0}$ due to frequency splitting. It is possible to express the drive frequency in terms of a deviation from $\omega_{0}$ termed the ‘gamma factor’ [24]:

(7) $$\gamma =\frac{\omega }{\omega_{0}}$$

The primary and secondary impedances $Z_{P}$ and $Z_{S}$ can also be defined in terms of *γ*:

(8a) $$Z_{1}=R_{1}+j\left(\omega L_{1}-\frac{1}{\omega C_{1}}\right)=R_{1}+j\omega L_{1}\left(1-\frac{1}{\gamma^{2}}\right)$$

(8b) $$Z_{2}=R_{2}+j\omega L_{2}+\frac{1/R_{L}-j\omega C_{2}}{1/R_{L}^{2}+{\left(\omega C_{2}\right)}^{2}}=R_{2}+\frac{R_{L}}{1+\alpha }+j\left(\omega L_{2}-\frac{\gamma^{2}R_{L}^{2}}{\omega L_{2}\left(1+\alpha \right)}\right)$$

where $\alpha ={\left(\omega C_{S}R_{L}\right)}^{2}$ as defined in [25]. By substituting the expressions in Equation (8) into Equation (6), and using the definition of Q-factor such that $Q_{P}=\omega L_{P}/R_{P}$ and $Q_{S}=\omega L_{S}/R_{S}$, the gain can be defined in terms of Q-factor, *α*, and *γ*:

(9) $$A\left(\gamma ,k\right)=\frac{-jk\sqrt{L_{S}/L_{P}}}{\left(\frac{1}{Q_{P}}+j\left(1-\frac{1}{\gamma^{2}}\right)\right)\left(\gamma^{2}\left(\frac{1}{Q_{S}\sqrt{\alpha }}-1+j\left(\frac{1}{Q_{S}}+\frac{1}{\sqrt{\alpha }}\right)\right)+1\right)+k^{2}\left(j\gamma^{2}+\frac{1}{\sqrt{\alpha }}\right)}$$

Equation (9) provides a convenient expression for the link gain for different values of *k*, $R_{L}$ (contained within *α*), and *γ*. Using the definition of gain in Equation (9), the effect of frequency splitting on the link gain can be considered.

### 2.2. Frequency Splitting in Overcoupled Inductive Links

Frequency splitting can be described as a deviation of the *link resonant frequency*, referred to from here on as the *optimum frequency* $\omega_{\text{opt}}$, from $\omega_{0}$. Frequency splitting occurs when an inductive link is *overcoupled*, i.e., when the coupling *k* is greater than the critical coupling $k_{\text{crit}}$. For $k=k_{\text{crit}}$, the link gain is maximised when the link is driven at the tuned frequency, i.e., $\omega_{\text{opt}}=\omega_{0}{|}_{k=k_{\text{crit}}}$ [26]. While determination of the exact splitting frequencies is unnecessary in this context, $k_{\text{crit}}$ for a given link must be identified, so that the overcoupled and undercoupled regions can be defined.

### 2.3. Relationship between Coupling and Coil Separation

When designing a pair of link coils to power an IMD, it is important to know the value of critical coupling $k_{\text{crit}}$, so that the over/undercoupled regions can be defined. $k_{\text{crit}}$ can be defined in terms of the Q-factors [24]:

(10) $$k_{\text{crit}}=\frac{1}{\sqrt{Q_{P}Q_{S}}}$$

$k_{\text{crit}},$ however, is not a value that is immediately applicable to a design procedure; the value of *k* for a given distance *d* between two coils depends strongly on the coil geometries. Additionally, to measure the coupling *k* between a pair of coupled coils requires a complex empirical test process [25]. The distance d, however, can be directly related to a design by comparing $d_{\text{crit}}$ with the expected implantation depth. In order to avoid this complex procedure for measuring *k*, it is convenient to translate from *k* to *d* using Equation (11) [27], which translates between *M* and *d* for given coil geometries (where $M=k\sqrt{L_{P}L_{S}}$):

(11a) $$M_{ij}=\frac{2\mu }{a}\sqrt{r_{i}r_{j}}\left[\left(1-\frac{a^{2}}{2}\right)K\left(a\right)-E\left(a\right)\right]$$

(11b) $$a=2\sqrt{\frac{r_{i}r_{j}}{{\left(r_{i}+r_{j}\right)}^{2}+d^{2}}}$$

(11c) $$M=g\sum_{i=1}^{N_{P}}\sum_{j=1}^{N_{S}}M_{ij}\left(r_{i},r_{j},d\right)$$

where $r_{i}$ and $r_{j}$ are turn radii of the primary and secondary coils, respectively, *μ* is the magnetic permeability of the surrounding material, K(a) and E(a) are complete elliptic integrals of the first and second kind, respectively, $N_{P}$ and $N_{S}$ are the number of primary turns and secondary turns, respectively, and *g* is a shape factor that must be experimentally determined (for printed square coils g=1.1 [27]).

### 2.4. Effects of Frequency Splitting

**Figure 2:**
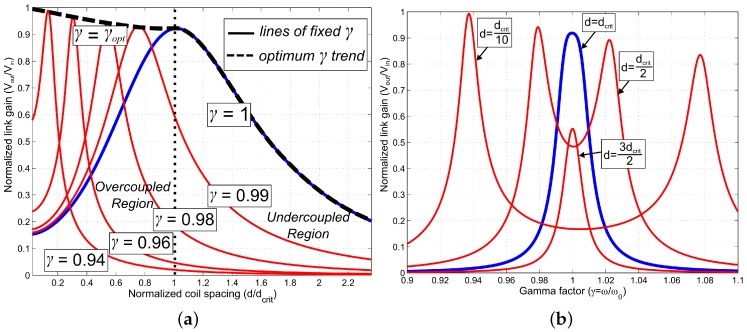
(**a**) Normalized link gain against normalized coil spacing and (**b**) normalized link gain against gamma factor, for the coil parameters in Table 1 with $R_{L}=100$ kΩ.

Figure 2 shows the profile of the link gain versus variations in coupling and frequency, as modelled by Equations (9) and (11), for the coils specified in Table 1.

Figure 2a shows normalized link gain vs. coil spacing distance. When driving at $\omega_{0}$ (γ=1), the gain is defined by a bell shaped curve with a peak at critical coupling ($d=d_{\text{crit}}$). As the distance between the coils moves above or below $d_{\text{crit}}$, a significant loss of gain occurs. In the undercoupled case ($d>d_{\text{crit}}$), the loss is due to a reduction in coupling *k*. In the overcoupled region ($d<d_{\text{crit}}$), the loss is due to frequency splitting, and can be compensated for by adjusting the input frequency of $V_{\text{in}}$ to the optimum frequency $\omega_{\text{opt}}$ ($\gamma_{\text{opt}}=\omega_{\text{opt}}/\omega_{0}$). For instance, in Figure 2a, if the coil spacing reduces to half the critical spacing, a 50% loss in gain occurs if the drive frequency is maintained at $\omega =\omega_{0}$. By reducing the driving frequency such that γ=0.96, the link gain increases by 5% compared to the value at $d_{\text{crit}}$, and increases by more than 100% for the same distance of $d/d_{\text{crit}}=0.5$. The $\gamma_{\text{opt}}$ trend shown in Figure 2a illustrates that by maintaining the driving frequency at $\omega_{\text{opt}}$ while $d<d_{\text{crit}}$, the link gain is maximised at a value equal to or greater than the gain when $d=d_{\text{crit}}$.

Figure 2b highlights how frequency splitting manifests at different coil spacing distances. When the link is critically coupled or undercoupled ($d\leq d_{\text{crit}}$), the gain function is again represented by a bell-shaped curve, with a peak at $\omega_{0}$ (γ=1). When the link is overcoupled, however, this single peak splits into a pair of peaks at frequencies either side of $\omega_{0}$, one of which represents the optimum frequency, $\omega_{\text{opt}}$ for $V_{\text{in}}$. This is generally the lower frequency peak, as there will be less impact from parasitic high frequency effects. The frequencies at which these peaks occur can be determined analytically [10].

### 2.5. Effects of $R_{L}$

The definition of $k_{\text{crit}}$ in Equation (10) is only accurate if $R_{L}$ is assumed to be infinite. This assumption holds up for some very low power implants [28]. However, it is more realistic to assume that $R_{L}\ll \infty$.

**Figure 3:**
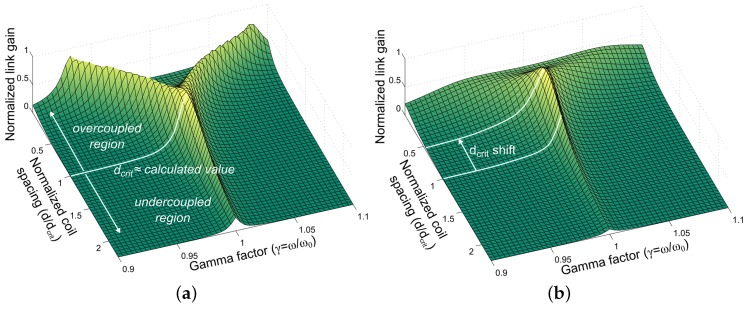
Link gain against variable distance and frequency for two distinctly different load resistances. (**a**) $R_{L}$ = 100 kΩ; (**b**) $R_{L}$ = 1 kΩ.

The effect of $R_{L}$ is to add more parallel loss at the receiver, reducing $Q_{S}$. The breakdown of Equation (10) is highlighted by the plots in Figure 3.

For very large resistances $R_{L}$, frequency splitting is prominent, and the calculated value of $d_{\text{crit}}$ is very close to the correct value. As this resistance approaches zero, splitting becomes less prominent, and the actual critical distance varies considerably from the calculated value: Figure 3b shows an error of approximately 50% compared with the value calculated from Equation (10).

To make Equation (10) more accurate when considering a loaded link, the way $Q_{S}$ is determined must include the additional parallel loss from $R_{L}$. If the parasitic secondary series loss $R_{S}$ is converted to a parallel loss, it can be considered in parallel with $R_{L}$ to find a ‘loaded Q’ value for $Q_{S}$, which accounts for the presence of $R_{L}$. Equation (12) provides a means of recalculating $Q_{S}$ to account for $R_{L}$:

(12) $$Q_{S(\text{loaded})}=\frac{R_{L}\left(Q_{S}+1/Q_{S}\right)}{\omega L_{S}\left(Q_{S}+1/Q_{S}\right)+R_{L}}\approx \frac{R_{L}Q_{S}}{\omega L_{S}Q_{S}+R_{L}}{|}_{Q_{S}>10}$$

Therefore, by replacing $Q_{S}$ in Equation (10) with $Q_{S(\text{loaded})}$ from Equation (12), it is possible to calculate much more accurate values for $k_{\text{crit}}$ and $d_{\text{crit}}$ when $R_{L}\ll \infty$.

### 2.6. Considerations for Tracking $\omega_{\mathrm{opt}}$

The results in Figure 2 and Figure 3 show that $\omega_{\text{opt}}$ for a given link depends strongly on both the coil spacing and link load. Seeing that the spacing and load are both variable factors, it is insufficient to assume nominal fixed values and calculate a nominal fixed value of *ω* if the goal is to maintain a maximised output voltage. It is necessary, therefore, to devise a system that is capable of tracking $\omega_{\text{opt}}$ regardless of changes in *d* and $R_{L}$. The system proposed in Section 3 achieves this by monitoring the phase difference between the primary current and voltage; allowing it to track $\omega_{\text{opt}}$ regardless of *d* and $R_{L}$.

## 3. Frequency Tracking System

A closed-loop transmitter system was designed to compensate for frequency splitting that occurs in the overcoupled region of inductive links. The aim of the system is to adjust the drive frequency so that the phase between the voltage and current in the primary side is close to 0${}^{\circ }$, ensuring operation at resonance.

**Figure 4:**
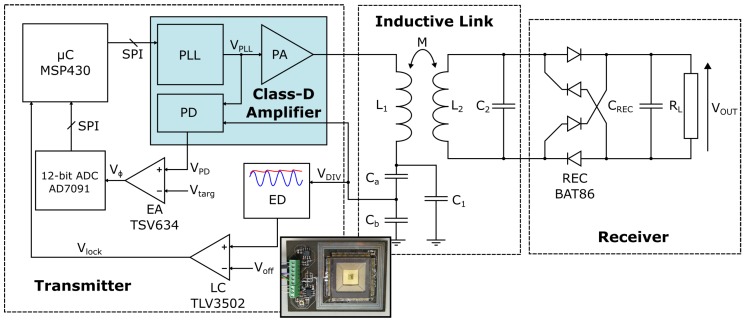
Architecture of the closed-loop OFT system. Inset: photo of the transmitter prototype. The prototype contains the primary side of the inductive link, ED, LC, EA, and the Class-D amplifier. The microcontroller and ADC are connected externally.

Figure 4 shows a block diagram of the system, which includes a Class-D amplifier chip [23]. This chip includes a high resolution phase-locked loop (PLL) for clock generation, a programmable delay-line for optimizing the drive signal dead time, a phase detector (PD) referenced against the PLL clock, and a Class-D output stage capable of an output power of up to 30 W (at a supply voltage of up to 30 V). The chip was fabricated in a 0.18 μm CMOS technology. The inductive link formed by $L_{1}$, $L_{2}$, $C_{1}$, and $C_{2}$ is driven by the power amplifier (PA). In theory, the high Q of the resonant tanks means that the link currents can be considered as sinusoidal at the fundamental harmonic of the PLL frequency. The AC signal induced in the secondary coil is converted to DC via a discrete Schottky diode rectifier bridge, smoothed by the capacitor $C_{\text{REC}}$, and delivered to the load $R_{L}$. The current in $L_{1}$ is monitored by using a capacitive divider formed from $C_{a}$ and $C_{b}$ that give the voltage $V_{\text{DIV}}$. The purpose of the divider is to reduce the load on the primary from the monitoring circuit, and protect the monitoring circuit from the high voltage that can develop in the primary. $V_{\text{DIV}}$ lags the primary current by $90^{\circ }$, and serves two purposes.

First, the amplitude of $V_{\text{DIV}}$ is checked using an envelope detector (ED) and a ‘lock comparator’ (LC) (TLV3502, Texas Instruments, Dallas, TX, United States), which compares the ED output level with a pre-set offset voltage $V_{\text{off}}$. The output of the LC, $V_{\text{lock}}$, is then fed to a microcontroller (MSP430, Texas Instruments). $V_{\text{lock}}$ is high when the link is close enough to resonance for the ED output to be higher than $V_{\text{off}}$; this allows room for calibration. Second, $V_{\text{DIV}}$ is fed to the on-chip PD for comparison with the clock signal, $V_{\text{PLL}}$, generated by the on-chip PLL. The output of the PD is applied to an error amplifier (EA), which compares it with a target error voltage, $V_{\text{targ}}$, which should represent $90^{\circ }$ phase difference between $V_{\text{DIV}}$ and $V_{\text{PLL}}$ when calibrated correctly. The resulting voltage from the EA, $V_{\phi }$, is digitized by a 12-bit analogue to digital converter (ADC) (AD7091, Analog Devices) and fed back to the microcontroller. The EA consists of a differential gain stage and a single ended integrating stage. The integrating stage at the end of the chain damps the transient response to prevent instability. By making use of $V_{\text{lock}}$, to confirm the link is near resonance, and $V_{\phi }$, to monitor the phase angle between the primary current and voltage, the microcontroller can track the link state and adjust the PLL frequency so it stays at $\omega_{\text{opt}}$. Section 3.1 and Section 3.2, respectively, describe the operation of the transmitter system and the specifics of the inductive link coils.

### 3.1. Transmitter System Operation

**Figure 5:**
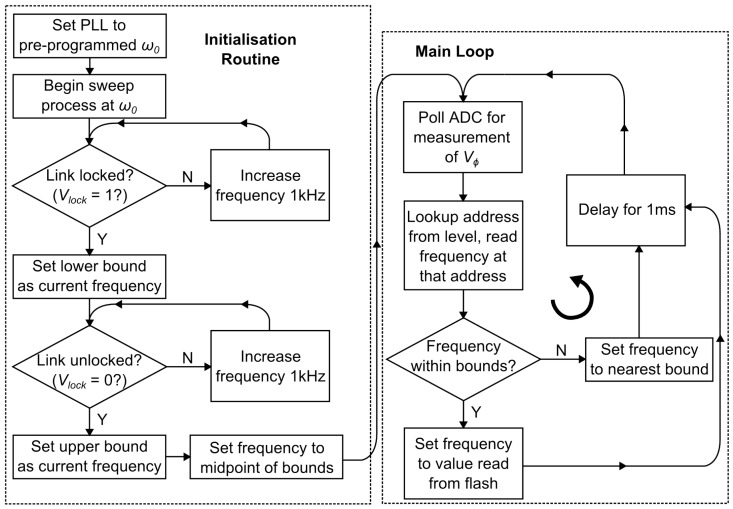
Flow diagram of the operation of the microcontroller control unit.

The operation of the transmitter system can be set to either fixed-frequency mode (at $\omega_{0}$) or OFT mode, where the input frequency should automatically be set to $\omega_{\text{opt}}$. When operating in OFT mode, the system can be considered as a control system employing negative feedback, with the feedback signals represented by $V_{\phi }$ and $V_{\text{lock}}$. The primary coil current is measured by using a capacitive divider formed of $C_{a}$ and $C_{b}$ ($C_{a}$ = 10 pF, $C_{b}$ = 33 pF), as part of the primary resonance capacitor $C_{1}$. The phase difference between the coil current and the PLL clock is measured by the on-chip PD [23]. The photo inset in Figure 4 shows an example of a miniaturised prototype of the transmitter system, used to confirm successful operation of the feedback system even in very close proximity to the link’s magnetic field. The feedback action of the circuit operates under the control of the microcontroller as detailed in the flow diagram in Figure 5.

Initially the driving frequency is set to $\omega_{0}$, which represents the resonant frequency of a specific coil pair at critical coupling. The PLL frequency is then varied either side of $\omega_{0}$ in 1 kHz steps to determine the working range of frequencies for the link. The upper and lower frequency bounds are then defined while the coils are brought together (recall that the frequency splitting phenomenon shows two peaks in the link gain curve either side of $\omega_{0}$, so only one is chosen). This process makes use of the feedback signal $V_{\text{lock}}$, so that checking whether the envelope of $V_{\text{DIV}}$ is greater than a specified offset $V_{\text{off}}$, determines if the link is close to resonance. After locating this working frequency range, the microcontroller enters the control loop routine (Main Loop in Figure 5). The error signal $V_{\phi }$ is read from the ADC, and translated by the microcontroller into a frequency code that updates the PLL frequency.

### 3.2. Coil Design and Implementation

The link was tested using square printed spiral coils, fabricated on a standard FR4 substrate, and optimised for 5 MHz operation. To obtain the design parameters for the coils, the iterative process described in [27] was employed through a script in MATLAB. Initial constraints were applied to the following parameters: implanted coil outer diameter $d_{o2}$, typical load $R_{L}$, working coil spacing *d*, and minimum PCB track spacing *s*. The constraints were $d_{o2}=d=$ 20 mm, $R_{L}=\text{10}\;\text{k}\Omega$, s=150μm. The constraint on spacing was a conservative estimate of the limitations of the available PCB fabrication facility, while the other constraints were common example values in the context of biomedical implant size, implantation depth, and power requirements [29]. The measured coil parameters of the fabricated coils are listed in Table 1.

## 4. Testing and Results

### 4.1. Test Procedure

The operation of the closed-loop system was tested with a bench setup, in which the printed coils were mounted on a jig. This provided control over the lateral displacement between the coils from 5 mm to 70 mm. The system efficiency was calculated in terms of the DC power drawn from the supply and the secondary voltage $V_{\text{OUT}}$ across the load $R_{L}$. Therefore, the efficiencies stated are *system* efficiencies, not link efficiencies.

Initially, the coils were aligned on the jig, and placed far enough apart to ensure power transfer in the undercoupled region (*d* = 70 mm); the value of $\omega_{0}$ could then be empirically determined through manual frequency adjustment. The distance between the coils was then manually adjusted between 5 mm and 50 mm in steps of 5 mm, and $V_{\text{OUT}}$ at the load was measured with OFT disabled and enabled. In a second test, the distance was varied as before, but the power supply was also manually adjusted such that $V_{\text{OUT}}$ = 10 V; the efficiency was recorded. In a third test, the link coils were fixed in an overcoupled position (*d* = 5 mm), and the load $R_{L}$ was varied; again $V_{\text{OUT}}$ and system efficiency were recorded, with OFT enabled and disabled.

### 4.2. Link Measurements

The operation of the OFT mode was tested at frequencies around the nominal 5 MHz, with the link parameters specified in Table 1, and a supply voltage of 5 V.

**Figure 6:**
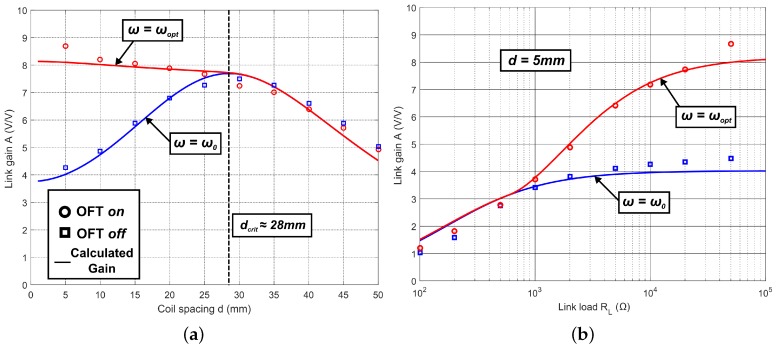
Link gain against changes in coil spacing *d* and link load $R_{L}$. Solid lines represent calculated behaviour, data points are experimentally determined. (**a**) Spacing *d* vs. Gain *A*, $R_{L}$ = 100 kΩ; (**b**) Load $R_{L}$ vs. Gain *A*, *d* = 5 mm.

Figure 6a shows the measured (data points) and calculated (solid lines) relationships between coil spacing and link gain when $R_{L}$ = 100 kΩ. In the overcoupled region, the OFT compensation scheme demonstrates an increase in link gain when compared with the fixed-frequency approach; the experimental data closely match the predicted results. The overcoupled region for the coils used in this study extends to a distance of approximately 28 mm, beyond which the trends converge as $\omega_{\text{opt}}\to \omega_{0}$. At *d* = 5 mm, there is a notable deviation in the experimental link gain from the calculated value. This deviation is attributed to capacitive coupling between the coils becoming significant, particularly given the relatively high values of $R_{L}$ used. Over the range *d* = 5 mm to 27 mm, the results show that OFT can provide a significant improvement in link gain in the overcoupled region, with measured gain improvements greater than twofold at *d* = 5 mm. This improvement in gain allows for reduction in the supply voltage at the transmitter, while still achieving the target $V_{\text{OUT}}$, allowing for reduced overall power consumption. Figure 6b shows the measured and calculated link gain versus variations in load resistance $R_{L}$ with OFT enabled/disabled. The OFT system can automatically compensate for changes in $R_{L}$ in the same way it can compensate for changes in coil coupling, with a twofold increase in link gain for OFT vs. fixed frequency when $R_{L}$ = 100 kΩ. The small discrepancy in measured results at *d* = 5 mm and $R_{L}$ = 100 kΩ in Figure 6b is due to mechanical and component tolerances in the link, resulting in small systematic errors between measurement runs. The convergence of the OFT on and off trends in Figure 6b occurs at approximately $R_{L}$ = 1 kΩ. This convergence indicates the point at which the load becomes significantly lower than the parallel loss resistance of the receiver coil, which was calculated, from the values in Table 1, to be approximately 1.85 kΩ. Figure 6b presents a similar deviation between measurement and prediction for $R_{L}>50\;\text{k}\Omega$ to that seen in Figure 6a. The fact that this deviation occurs only at the highest load values is further evidence that it is the result of stray capacitive coupling effects.

**Figure 7:**
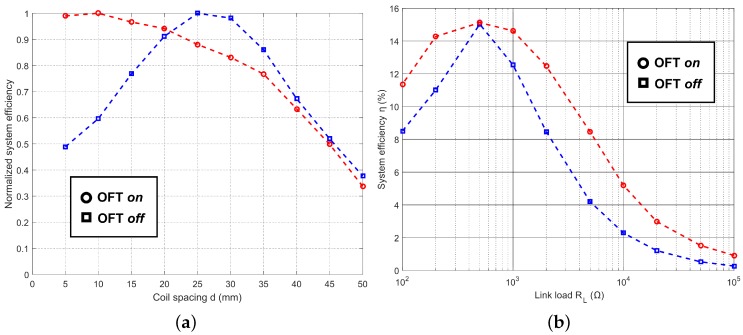
Plots to show experimental efficiencies against variations in load and coil spacing, with OFT enabled and disabled. (**a**) Normalized efficiency vs. spacing, $R_{L}=100\;\text{k}\Omega$; and (**b**) system efficiency vs. load, d=5mm.

The benefit of the OFT compensation scheme is more evident with the aid of Figure 7a, which shows the difference in normalized measured efficiency with and without OFT, while manually varying the supply to the transmitter PA in order to maintain a target $V_{\text{OUT}}$ = 10 V. The gain in system efficiency using OFT is significant in the overcoupled region, doubling at the smallest distance of 5 mm. Figure 7b shows the direct effect of OFT compensation on the efficiency of the system against changes in $R_{L}$. Figure 7b illustrates that by employing OFT, the system efficiency can be increased for almost all load values. Under specific link conditions, where $R_{L}=500\;\Omega$ and d=5mm, $\omega_{\text{opt}}$ was found to be equal to $\omega_{0}$, and so the overall system efficiency was equal for OFT on and off.

Figure 6 and Figure 7 together show that while there are some gains in system efficiency, the primary benefit of OFT is that it maintains a more constant output voltage at the receiver than the fixed frequency mode.

### 4.3. Real-Time System Operation

**Figure 8:**
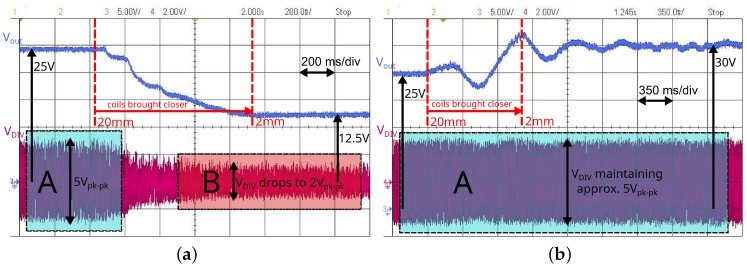
Scope screenshots to illustrate variations in $V_{\text{OUT}}$ in fixed mode and OFT mode as the coils are brought closer together. (**a**) OFT disabled; (**b**) OFT enabled.

Figure 8 shows the transient operation of the system with OFT disabled (Figure 8a) and enabled (Figure 8b). In the former case, the coils are placed at a distance of 20 mm, and the power supply is regulated to obtain 25 V across the load (100 kΩ). If the coils are brought closer to a distance of 5 mm, the voltage across the load will drop to 12.5 V (in accordance with Figure 6a). The inset sections labelled A and B in Figure 8a show the profile of $V_{\text{DIV}}$, which represents the current in the primary coil. The amplitude of $V_{\text{DIV}}$ drops from $5\;{\text{V}}_{\text{pk-pk}}$ to $2\;{\text{V}}_{\text{pk-pk}}$ when the coils are moved closer with OFT disabled. This drop in the amplitude will track the optimum frequency to keep the load voltage maximised. As the coils are brought closer together, the load voltage increases to 30 V after a short settling period. The ringing visible in Figure 8b occurs due to a combination of mechanical vibration of the coil displacement jig and overshoot in the control circuit. This behaviour is in accordance with the results in Figure 6a, where the gain increases as the link spacing decreases when OFT is enabled.

**Figure 9:**
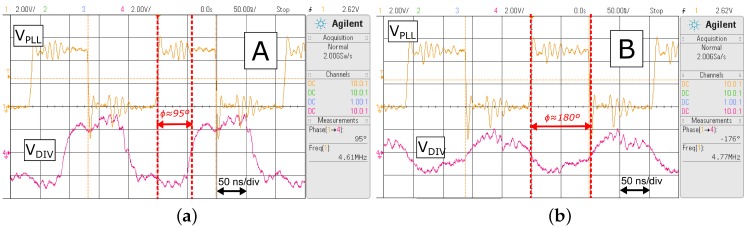
Close-up view of the labelled areas A and B in Figure 8, illustrating the difference between $V_{\text{PLL}}$ and $V_{\text{DIV}}$ while near resonance and far from resonance. (**a**) Near resonance ($\phi \approx 90^{\circ }$); (**b**) Far from resonance ($\phi \approx 180^{\circ }$).

Figure 9 shows a zoomed version of the inset sections labelled A and B in Figure 8. This highlights the variation in phase difference between $V_{\text{DIV}}$ and $V_{\text{PLL}}$ as the link is brought out of resonance by reducing the distance between the coils. The traces in Figure 9a show a phase difference *ϕ* of $95^{\circ }$ between $V_{\text{DIV}}$ and $V_{\text{PLL}}$, and this indicates resonance in the link; when the phase between $V_{\text{DIV}}$ and $V_{\text{PLL}}$ is $90^{\circ }$, the phase between the primary current and primary voltage is at $0^{\circ }$. The traces in Figure 9b show the effect of driving the link in a fixed mode such that $\omega \neq \omega_{\text{opt}}$. This manifests in a phase difference of $\phi =180^{\circ }$ and a drop in the amplitude of $V_{\text{DIV}}$ as the coils are moved closer together.

Together Figure 8 and Figure 9 show how, by employing OFT, the output voltage is maintained at a high level even as the coils are displaced.

## 5. Conclusions

This paper has presented the implementation and operation of a closed-loop OFT system capable of compensating for frequency splitting and therefore improving the resilience and system efficiency of inductive WPT systems. The system requires no variable capacitors or inductors, and is suitable for compact systems where space is valuable, such as for IMDs. The system is capable of adapting to changes in coil coupling and load conditions, maintaining a constant output voltage and improved system efficiency compared to fixed frequency equivalents.

Measurements have shown close matching with the predicted behaviour of the system. Discrepancies were only observed at very small distances between the coils, where capacitative coupling becomes a relevant factor. These effects are currently under analytical and experimental investigation. This system is suited to powering implanted medical devices, particularly those in which the receiver moves significantly with respect to the transmitter, and has time variant power requirements, such as in endoscopic capsules [30]. This approach could also be useful in the case of distributed implants [2], where the distances between the transmitter and the receivers can be quite variable. Since OFT requires no back-telemetry to operate, this work is suitable for systems with very little complexity at the receiver, e.g. for direct inductive stimulation [31]. Additionally, in the case of implants employing flexible coils, the proposed OFT system would be capable of adapting to the altered link impedance presented by coil flexion [16] as a result of its ability to drive across a wide range of frequencies. OFT could also be used to interrogate remote units to establish the deviation in their true resonant frequencies from the designed value, or operate multiple receivers tuned at different frequencies [32]. Such an interrogation technique could also be used to confirm each receiver’s post-implantation optimum frequency.

Table 2 provides some recent examples of comparable biomedical WPT systems to contrast with the work presented here, highlighting the ability of this work to compensate for load and spacing changes, regardless of component variations, without requiring any data telemetry.

## Figures and Tables

**Table 1:** Measured coil parameters for operation at 5 MHz.

		Tx Coil	Rx Coil
Outer Diameter	$d_{o}$	64.7 mm	20.0 mm
Inner Diameter	$d_{i}$	37.2 mm	4.7 mm
Number of Turns	*n*	12	13
Inductance	*L*	10.150 μH	2.255 μH
Q-factor	*Q*	47.5	26.0

**Table 2:** Comparison of this work to some recent examples of comparable biomedical WPT systems.

	[13]	[12]	[19]	[31]	This Work
Operating Frequency	1 MHz	13.56 MHz	13.56 MHz	10 MHz	**Variable (4.5–5.5 MHz)**
Effective Range	15 mm	20 mm	20 mm	N/A	**30 mm**
Compensation Parameter	Supply Voltage	Supply Voltage	$C_{1}$, $C_{2}$, and Supply Voltage	None	**Drive Frequency**
System Efficiency (Best)	65.8%	14%	14%	N/A	**15%**
Component Mismatch Insensitive	No	No	Yes	No	**Yes**
Passive Implant Compatible	No	No	No	Yes	**Yes**

## Abbreviations

The following abbreviations are used in this manuscript:

WPT: Wireless Power Transfer
IMD: Implanted Medical Device
OFT: Optimum Frequency Tracking
PA: Power Amplifier
ED: Envelope Detector
PD: Phase Detector
LC: Lock Comparator
PLL: Phase-Locked Loop
EA: Error Amplifier
ADC: Analogue to Digital Converter
